# Immunosuppressive Mechanisms of Malignant Gliomas: Parallels at Non-CNS Sites

**DOI:** 10.3389/fonc.2015.00153

**Published:** 2015-07-06

**Authors:** Powell Perng, Michael Lim

**Affiliations:** ^1^Department of Neurosurgery, School of Medicine, Johns Hopkins University, Baltimore, MD, USA

**Keywords:** glioblastoma, tumor immunotherapy, cancer immunotherapy, cancer immunosuppression, glioma, immune privilege

## Abstract

The central nervous system (CNS) possesses powerful local and global immunosuppressive capabilities that modulate unwanted inflammatory reactions in nervous tissue. These same immune-modulatory mechanisms are also co-opted by malignant brain tumors and pose a formidable challenge to brain tumor immunotherapy. Routes by which malignant gliomas coordinate immunosuppression include the mechanical and functional barriers of the CNS; immunosuppressive cytokines and catabolites; immune checkpoint molecules; tumor-infiltrating immune cells; and suppressor immune cells. The challenges to overcoming tumor-induced immunosuppression, however, are not unique to the brain, and several analogous immunosuppressive mechanisms also exist for primary tumors outside of the CNS. Ultimately, the immune responses in the CNS are linked and complementary to immune processes in the periphery, and advances in tumor immunotherapy in peripheral sites may therefore illuminate novel approaches to brain tumor immunotherapy, and vice versa.

## Part I: Introduction

Contrary to common perceptions of central nervous system (CNS) “immune privilege,” the brain can in fact elicit vigorous immune-stimulatory as well as immunosuppressive responses, the determinants of which are highly contextual. Understanding the determinants and mechanisms of both the stimulatory and suppressive responses may help elucidate novel immune-based strategies for brain tumor immunotherapy. In this review, we will discuss routes of glioma-mediated immunosuppression, including mechanical and functional barriers of the CNS, immunosuppressive cytokines, immune checkpoint molecules, tumor-infiltrating immune cells, and suppressor immune cells (Table 1). In addition, we will look to analogous immune-modulatory mechanisms observed in other sites of the body, as discoveries made at CNS and non-CNS sites are ultimately complementary and equally relevant to therapeutic development for tumors at all sites (1).

**Table 1:** **Key examples of immune-modulatory mechanisms shared between malignant gliomas and non-CNS tumors**.

	Malignant gliomas	Non-CNS tumor
**TUMOR ANTIGEN PRESENTATION**		
Antigen-presenting cells	Glioma-associated microglia and/or macrophages (2–4); DCs (5); B lymphocytes (6); possibly pericytes (7)	DCs (8–11); tumor-associated macrophages (12–14); B lymphocytes (6); possibly pericytes (7)
Location of antigen presentation	Brain parenchyma and/or tumor mass (2–4); tumor-draining lymph nodes (15–17)	Tumor-draining lymph nodes (18–20); tumor mass (21)
Routes of antigen egress from tumor	Fluid drainage (22, 23); migrating DCs less likely (24–27)	Migrating DCs (28–30) and/or fluid drainage (31)
**IMMUNOSUPPRESSIVE CYTOKINES**		
IL-10		
Sources	Glioma-associated macrophages and microglia (32)	Tumor cells (33, 34) and/or tumor-associated macrophages (35)
Actions	Immunosuppression (various) (36, 37); context-dependent pro-inflammatory and anti-tumor actions (38, 39)	Immunosuppression (various) (40); anti-angiogenesis (41), anti-metastatic (41), anti-tumor (42–44); anti-inflammatory (45)
TGF-β		
Sources	Glioma cells (46–48) and glioma-associated immune cells (49)	Tumor cells and tumor-associated immune cells (49–51)
Actions	Immunosuppression (various) (52); angiogenesis (53); maintains glioma stem cell populations (54); glioma cell autocrine proliferation (55); pro-invasion (56)	Immunosuppression (various) (50, 57, 58); tumor suppression (50, 57–59)
**INDOLAMINE 2,3-DIOXYGENASE (IDO)**		
Sources	Glioma cells and tumor-associated immune cells (60–62)	Tumor cells, tumor-associated immune cells, and endothelial cells (62–65)
Actions	Immunosuppression (various) (66); expansion of Treg population (67, 68)	Immunosuppression (various) (66); expansion of Treg population (67, 68)
**REGULATORY T LYMPHOCYTES**		
Predominant Treg type	nTreg (69) more than iTreg	nTreg more than iTreg (70)
Relevant Treg recruitment factors	CCL22 (71–73), CCL2 (71, 74, 75)	CCL2, CCL22 (76–81), CCL17 (81); CCL3, CCL4 (82); CCL5 (83)
**TUMOR-ASSOCIATED MYELOID CELLS**		
Types	Microglia (84, 85), macrophages (86), MDSCs (87)	Macrophages (88), MDSCs (89, 90)
Actions	Immunosuppression (various) (91–95); tumor invasion (96); tumor proliferation (97, 98)	Immunosuppression (various) (99); tumor invasion (100); tumor proliferation (100)
**PD-1/PD-L1 IMMUNE CHECKPOINT**		
Sources	Glioma cells (101); microglia (102); glioma-associated macrophages (103); neurons in tumor-adjacent brain tissue (104)	Tumor cells (105–109), tumor-associated macrophages (100, 110, 111); healthy tissue (112–114)
Relevant signaling pathways	PI3K/mTOR (101)	PI3K/mTOR (106, 108, 115, 116), MyD88/TRAF6 (117), MEK/ERK (117)
Actions	Immunosuppression, esp. via T cell suppression (118, 119); induction of glioma cell death (104)	Immunosuppression, esp. via T cell suppression (120, 121)

## Part II: The CNS Immune Environment

The notion of “immune privilege” has long been ascribed to tissues wherein the immunological responsiveness is ostensibly blunted or modified (122). Early experimental observations that the brain lacked traditional lymphatic systems, contained few, if any, professional antigen-presenting cells (APCs), and mounted anemic immune responses against foreign antigens bolstered the theory that the brain was an “immunologically privileged” tissue. It is now apparent that the CNS is in fact capable of coordinating robust immune responses with the innate and adaptive immune systems, and that the immunological reactivity of the CNS is a mutable rather than an absolute state. Moreover, several of the structural and functional immunoregulatory features of the CNS that aid in dampening local immune responses are also reflected within other organs of the body. Therefore, the traditional notion of CNS “immune privilege” has become an imprecise characterization of the CNS immune environment, which is a more rightfully a highly contextual rather than an absolutely impregnable system.

### Reframing the CNS immune environment

In recent decades, the consensus view of the CNS immune environment has shifted from one in which the blood–brain barrier (BBB) serves as a static barrier against the exchange of cells and soluble molecules into one in which egress and entry are dynamically regulated, often by mechanisms observed in other organ systems. During inflammation, immune cells migrate into the CNS parenchyma following dynamic gradients of chemotactic cues, including IFN-γ inducible cytokines (123, 124), α and β integrins (125), and matrix metalloproteinases (126), which also play key roles in leukocyte trafficking in peripheral tissues (127). Similarly, it has been postulated that soluble immune effectors, such as immunoglobulins (128), might also cross the BBB. One possibility is by way of carrier-mediated transporters (129, 130). For example, FcRn, a ubiquitous immunoglobulin receptor expressed by a wide variety of tissues, can mediate Ig transport across tissue barriers (131, 132). Although the routes by which Igs enter the CNS parenchyma is yet unknown, it has been postulated that FcRn, which is highly expressed on cerebral vessels (131), may play a key role in facilitating Ig entry into the CNS, as in other tissues (133).

Whereas the absence of traditional lymphatic systems was once heralded as evidence that the CNS was immunologically inert (134), it is now abundantly clear that soluble antigens routinely egress the CNS and reach the peripheral lymph nodes. *In vivo* tracer studies have demonstrated that CNS antigens drain via cerebrospinal fluid across the cribiform plate and into the nasal sub-mucosa (135). A separate pathway by which antigens travel to the cervical lymph nodes (CLNs) via the Virchow–Robin perivascular spaces within walls of the cerebral arteries has also been described (22, 23). Indeed, during homeostatic conditions, antigens from the CNS are continuously sampled by DCs in the peripheral lymph nodes in the same fashion as antigens that arise from other sites (15). A more thorough discussion regarding antigen presentation in the CNS and peripheral tissues is provided in the next section of this review.

Lastly, although the entirety of CNS is often presumed to share the same immunological features, the relative absence of immune cells under homeostatic conditions is more accurately an attribute of the CNS parenchyma proper (127). At resting state, CSF-drained spaces, including the choroid plexus, leptomeninges, ventricles, and perivascular spaces, contain professional APCs and respond to foreign antigens in the same manner as organs do outside of the CNS (127, 136). By comparison, the parenchyma proper is generally devoid of peripheral immune cells and is maintained in a quiescent state by mechanical obstacles of the endothelial BBB (127). Obstacles against leukocyte entry include the CSF-drained Virchow–Robin perivascular space situated behind the endothelium, as well as the glia limitans, a wall of palisading astrocyte foot processes located between the perivascular space and CNS parenchyma (137). Aside from forming a second mechanical barrier against immune cells, the foot processes also express death ligand FasL/CD95L (138), which induces apoptosis in Fas-expressing T cells and arrests the inflammatory process. Accordingly, the vast majority of inflammatory cells that cross into the Virchow–Robin spaces during homeostatic states are retained in the perivascular space and never proceed past the glia limitans (127, 139). Inflammation and disease, however, can compromise the integrity of the BBB, thereby permitting circulating immune cells to infiltrate the parenchyma in significant numbers (136).

Hence, although the precise mechanisms underlying how and when the CNS coordinates immune responses remain to be clarified, there is accumulating evidence that several of the immunoregulatory features observed in the brain are shared by other tissues in the body as well. Baseline FasL expression, for example, is not unique to cerebral astrocytes but is also a feature in multiple peripheral tissues where immune homeostasis is favored, including lymphoid tissue, hepatocytes, testis, striated muscle, as well as certain glandular tissues (140–142). Blood–tissue barriers formed by intercellular tight junctions exist in the testis as they do in the CNS, and multiple organs, including the brain, liver, and gastrointestinal tract, secrete immune-modulatory cytokines that increase regulatory T cell expression and induce local immune tolerance (122). Therapeutic developments designed to overcome the immune-regulatory mechanisms of the BBB may therefore arise from discoveries made in the brain as well as findings made at other sites.

## Part III: Tumor Antigen Presentation

Classically, extracellular antigens are captured at the cell surface, endocytosed, and presented on MHC class II molecules to CD4+ T-lymphocytes by specialized APCs (143). By comparison, endogenous antigens are processed in the rough endoplasmic reticulum of nearly all cell types and subsequently presented on MHC class I molecules to CD8+ T lymphocytes (144). Presentation of tumor antigens, however, is thought to involve a third process, termed “cross-presentation,” whereby exogenous tumor antigens, scavenged from dying tumor cells, are presented on MHC Class I molecules to CD8+ T-lymphocytes, thereby directing the adaptive immune response toward malignant cells (145).

In peripheral sites, activation of tumor antigen-specific T cells is believed to take place within secondary lymphoid tissue, mediated by bone marrow-derived DCs via cross-presentation (145). Far less is known, however, regarding the process of priming T-cells against CNS tumor antigens (146). In particular, it remains unclear whether the anti-tumor immune response is initiated locally within the brain or peripherally in the body. The provenances of these processes have clear implications for brain tumor immunotherapies, such as dendritic cell-based vaccines (147, 148), that aim to exploit tumor antigen presentation to augment tumor immunity.

### Tumor antigen presentation: CNS

Whether CNS tumor antigen presentation occurs within the brain or outside of it remains unclear, though the presence of APCs within the brain supports the hypothesis that presentation begins locally. Because of their strategic position behind the BBB and their essential role in CNS innate immunity, microglia are often charged with being the primary APCs for intracranial antigen presentation. The data show that microglia have the capacity to cross-present tumor antigens to CD8+ T cells via MHC class I *in vitro* (2–4) and *in vivo* (2, 4). Employing a murine model in which whole-body radiation was used to eliminate peripheral and CNS-associated APCs (e.g., DCs and macrophages in the perivascular spaces), Jarry et al. recently demonstrated that microglia could successfully cross-present intra-cerebrally injected OVA antigen to naïve CD8+ T cells *in vivo* (4), strengthening prior data (2).

Tumor-infiltrating DCs may also play a key role in glioma antigen presentation (146). The data indicate that DCs cross-present OVA antigen more efficiently than adult microglia, eliciting greater quantities of IL-2 and IFN-γ production from CD8+ T cells than microglia (2). Similarly, Jarry et al. reported that CD8+ T cell activation was more efficient in non-irradiated mice, which contained CNS-associated DCs along with microglia, than in irradiated mice, which contained solely microglia (4). Especially given that flow-cytometry (FACS) markers used to identify DCs lack the specificity necessary for distinguishing between DCs and activated microglia (5), APC activity may be falsely attributed to microglia in many cases.

Whether microglia and tumor-infiltrating DCs can successfully activate CD8+ T cells in the setting of malignant brain tumors, however, is uncertain. Current data suggest that microglia lose their capacity to express MHC molecules in the context of high-grade gliomas (3, 149, 150), likely due to the high levels of immunosuppressive cytokines, such as TGF-β, IL-10, and PGE2, within the glioma microenvironment (151, 152). Even after removing microglia from the glioma environment, the ability for the microglia to upregulate MHC expression following stimulation was substantially depressed compared to normal brain microglia (153). Moreover, in the presence of glioma cells, microglial production of pro-inflammatory cytokine TNF-α is suppressed by as much as 50% compared to normal microglia, and activation of STAT3 transcription factor and secretion of immunosuppressive IL-10, both of which modulate immunosuppression, are greatly upregulated (154). Similarly, IL-10 has also been shown to inhibit DC maturation and maintain DCs in a tolerogenic state (155). These data suggest that malignant gliomas may skew APCs toward immunosuppressive phenotypes and hinder effective tumor antigen presentation within the brain. *In vivo* tumor models are needed to assess whether the APC capacities of microglia and DCs are in fact compromised in the glioma parenchyma and microenvironment.

Aside from microglia and DCs, tumor-associated macrophages (TAMs), B lymphocytes, and vascular pericytes may provide other cellular sources for CNS tumor antigen presentation. TAMs, which infiltrate gliomas in large numbers and possess cross-presentation capabilities (156), are thought to actually outnumber microglia (86) within the tumor mass. With regard to antigen-presentation capacity, data from a murine model of multiple sclerosis suggest that, compared to microglia, CNS-infiltrating macrophages are more highly activated and stimulate greater T cell proliferation *in vitro* (157). To our knowledge, however, no study to date has explicitly compared tumor antigen cross-presentation capacity of microglia to TAMs, likely due to limitations in reliably distinguishing microglia from TAMs within gliomas (158). Given that microglia and TAMs can both be polarized toward immunosuppressive M2-like phenotypes by the same sets of glioma-derived cytokines (159), it is possible that antigen-presenting capacity of microglia and TAMs are similarly impaired by the immunosuppressive glioma microenvironment.

B cells, which can function as efficient APCs outside of the CNS (6), are also believed to play a vital role in tumor antigen presentation in gliomas. Using a murine glioma model along with separate adenoviral vectors (Ad) encoding herpes simplex virus type I thymidine kinase (Ad-TK) and *fms-*like tyrosine kinase 3 ligand (Ad-Flt3L), which were used to kill tumor cells and recruit APCs to the microenvironment, Candolfi et al. showed that treatment with Ad-TK + Ad-Flt3L produced long-term survivors in 60% of WT mice but 0% in B-cell depleted mice (160). Moreover, when Ad-TK + Ad-Flt3L was administered to mice lacking transcriptional repressor Blimp-1, the absence of which causes arrest of terminal differentiation of B cells into antibody-producing plasma cells, Blimp-1-negative mice produced identical numbers of long-term survivors as WT mice, suggesting that tumor regression occurred irrespective of whether anti-tumor antibodies were generated (160). Lastly, in Ad-TK + Ad-flt3L-treated mice, the accumulation of antigen-bearing activated B cells within tumor-draining lymph nodes (TDLNs) along with evidence that the activated B cells were capable of stimulating CD8+ T cell proliferation *in vitro* were strong clues that B cells can cross-prime CD8+ T cells against glioma antigens and thereby orchestrate glioma regression (160).

Pericytes, which are perivascular cells that classically modulate blood flow, vessel permeability, and vessel remodeling at arterioles, venules, and capillaries, have also been shown to possess phagocyte and antigen-presentation capacity (7). Indeed, Peiper et al. recently reported that brain capillary pericytes, which are exquisitely sensitive to inflammatory cytokines, increase phagocytic activity and MHC class II expression when stimulated by TNF-α or IFN-γ (161). Key questions surrounding whether pericytes posses cross-presentation capacity and how the glioma microenvironment influences pericyte antigen-presentation ability remain to be answered. There are data from non-CNS tumor models, however, that suggest tumor-derived vascular pericytes may play an overall immunosuppressive role, and APC activity may therefore be impaired (162).

Interestingly, recent work by Thompson et al. illuminated that priming and differentiation of naïve CD8+ T cells can occur within tumor masses, irrespective of intratumoral APCs or TDLNs (21). It has been shown that prolonged TCR stimulation in the absence of CD28 co-stimulation might alone be sufficient for activating T cells (163), and high densities of tumor antigens within tumor masses may thereby provide a prolonged and powerful enough of a stimulus to activate T cells irrespective of APCs (21). Although these specific experiments involved melanoma tumors in non-CNS sites, there is also evidence that brain tumors themselves support terminal differentiation of CD8+ T cells (164). Therefore, the findings by Thompson et al. may yet find parallels in malignant brain tumors.

It is also possible, however, that presentation of brain tumor antigens occurs within peripheral lymphoid tissues outside of the CNS (16). Routes by which CNS antigens drain to the nasal mucosa and CLNs via CSF and/or perivascular spaces have been well described (22, 23), and recent evidence indicates that CNS antigens are continuously sampled in peripheral lymphoid tissue by DCs (15). Using intra-cerebral (IC) injections of fluorescent microspheres and OVA antigen in a mouse model, Walter et al. showed (1) that IC antigens preferentially accumulated in CLNs, and (2) that expansion of OVA-specific CD8+ T cells occurred within CLNs 2 days prior to their appearance in the brain, suggesting that cross-presentation occurs in the CLNs and not within the brain parenchyma (16). In a separate study, Okamoto et al. showed that 2 weeks following cerebral implantation of glioma tumors in rats, activated CD4+ and CD8+ T cells appeared exclusively within the CLNs, and their accumulation coincided temporally with T-cell infiltration into the tumor (17). Collectively, these data suggest that presentation of CNS tumor antigens may initiate in lymphoid tissue outside of the CNS.

Finally, it is also conceivable that priming the anti-tumor immune response involves processes both within and outside of the CNS. Transferring pre-activated tumor-specific CD8+ T cells into glioma-bearing mice, Masson et al. demonstrated that further phenotypic differentiation of tumor-specific CD8+ T cells occurs within the tumor mass (164). Compared to the pre-activated tumor-specific CD8+ T cells, tumor-infiltrating CD8+ T cells showed enhanced expression of IFN-γ, granzyme B, and α_E_(CD103)β_7_ integrin, the latter of which was found to be important for T-cell retention within the brain (164). Further analysis of human glioma tissue revealed similar differentiation patterns, with 20–57% of tumor-infiltrating CD8+ T cells expressing α_E_(CD103)β_7_ integrin compared to fewer than 5% of CD8+ T cells in peripheral blood (164). Consistent with that of murine tissue, approximately 60% of α_E_(CD103)β_7_-expressing CD8+ T cells in human glioma tissue also co-expressed granzyme B (164). It has been hypothesized that locally secreted TGF-β, which induces α_E_(CD103)β_7_ expression in non-CNS sites (165), may also moderate α_E_(CD103)β_7_ expression on T cells within gliomas (164). Further work is needed to evaluate how the glioma microenvironment initiates and/or shapes the effector immune response.

### Tumor antigen presentation: Non-CNS sites

In comparison to CNS tissues, there is a greater degree of clarity regarding the process of tumor antigen presentation in non-CNS tissues, though several aspects remain under contention. A preponderance of data indicate that presentation of tumor antigens occurs within the TDLNs, where resident DCs have been shown to play the key roles in priming naïve T cells (18–20). Additionally, several experiments have demonstrated that resident DCs within TDLNs can indeed cross-present tumor antigens to CD8+ T cells *in vivo* (8–10). Though macrophages are also endowed with cross-presentation capacities, they are substantially less efficient than DCs at priming CD8+ T cells (12–14). In the absence of convincing data supporting the primacy of alternative mechanisms, DCs have been presumed as the main APCs for cross-priming tumor-directed CTLs at non-CNS sites.

Further investigation, however, is needed to clarify the precise roles as well as cross-presentation capacities of DC subsets in tumor antigen presentation, as experimental models show that DC phenotypes can vary greatly depending on tissue and/or antigen type. DCs in murine lung tissue, for example, display CD103+ CD11b− and CD103− CD11b^hi^ phenotypes while colonic DCs exhibit a predominately CD103−CD11b+ phenotype. Human liver harbors myeloid-derived CD1c+ DCs (166) while human renal tissue contains a greater portion of lymphoid-derived or plasmacytoid DCs (pDCs) than conventional myeloid-derived DCs (167). During CNS inflammation, the brain parenchyma is heavily infiltrated with DCs displaying CD11c+ phenotypes (18). Of note, a recent analysis of three resident DC subsets from human tonsil lymphoid tissue demonstrated that all subsets were capable of cross-priming CD8+ T cells with high efficiency (11).

Several animal studies have also illustrated that distinct DC subsets may mediate antigen presentation depending on type and location of antigen exposure (168–173). For example, whereas CD8α+ CD11b− DCs mediated cross-presentation of OVA antigen in the spleen, CD8α− CD11b+ DCs were responsible for OVA cross-presentation in the mesenteric lymph nodes (173). Analysis of circulating DCs in patients with NSCLC and breast cancer further revealed disparities in the proportion of pDCs to conventional myeloid-lineage DCs between the two malignancies, suggesting that tumor type may influence DC phenotypes (174). Further work is needed to evaluate the roles of phenotypic DC subsets in tumor antigen presentation as well as how tumors may influence phenotypic differentiation, as these are all important considerations for developing tailored immunotherapeutic interventions for various tumor sites (11).

As with the CNS, B lymphocytes and vascular pericytes may also participate in tumor antigen presentation at non-CNS sites. In fact, it has been shown that in mice that have been immunized against specific protein antigens, CD40 ligand-activated B lymphocytes traffic to secondary lymphoid organs and present peptide antigens to naïve T cells with comparable efficacy to DCs (175). Recently, B lymphocytes pre-loaded with specific tumor antigens were used successfully as a source of APCs for tumor eradication in an experimental model (176). As with the CNS, pericytes are potential sources for perivascular phagocyte activity at non-CNS sites (7). Further work is needed to determine whether pericytes associated with non-CNS tumors contribute to tumor antigen presentation and/or immune evasion.

The manner in which tumor antigens reach TDLNs at non-CNS sites also requires further clarification. Traditionally, it has been assumed that migrating DCs carry tumor antigens from the tumor site to TDLNs, where antigens are then transferred to resident DCs for subsequent T-cell priming (28–30). Evidence from viral models, wherein DCs carried antigens from the site of injection to draining lymph nodes for CTL activation, lent credence to the theory (29, 30, 177–179). The need for migrating DCs for antigen presentation in peripheral tissues was also a point of distinction between non-CNS and CNS tissues, where a preponderance of data suggested that intra-parenchymal DCs do not migrate to the CLNs in substantial numbers (24–27).

Recent evidence, however, has challenged the role of migrating DCs in tumor antigen presentation. Findings from several studies suggest that the immunosuppressive milieu of the tumor microenvironment may in fact hinder DC function and migration from peripheral tissues (180–184). IL-10, for example, which is produced by a number of tumors, prevents DC maturation and suppresses DC antigen-presenting capabilities (185). A recent study by McDonnell et al. reported that cross-presentation of tumor antigens within TDLNs was dependent on the continuous drainage of tumor antigens from the tumor site rather than DC migration (31), as is the case with CNS tissue. As previously discussed, Thompson et al., who described that priming of CTLs could occur within tumor masses themselves, raises the possibility that DCs altogether may be unnecessary for activating T cells (21). The high density of tumor antigens within the tumor parenchyma may provide sufficient stimulus for T-cell receptor (TCR) activation (21). Therefore, the cross-presentation of tumor antigens in peripheral tissues may in fact share commonalities with that of the CNS.

#### Antigen Presentation and Therapeutic Implications

In aggregate, these data show that much is still unknown regarding whether antigen-specific T cells, directed against CNS tumors, are primed locally in the CNS or peripherally in non-CNS sites. However, the data do speak strongly to the notion that priming tumor-specific T cells may, at least in part, occur within the body, emphasizing the need to evaluate anti-tumor immune responses directed at CNS tumors within a global context. Whether initial tumor antigen presentation occurs in the brain or in the body, for example, could have significant design implications for whether vaccine-based glioma therapies are designed for intracranial or peripheral administration.

Recent progress in evaluating tumor antigen presentation in the body has also identified shared features with the CNS. Similar to brain, peripheral tissues may also depend upon fluid drainage of tumor antigens to TDLNs rather than migrating DCs for the purpose of priming tumor-specific T-cells (31). Tumor-associated immunosuppressive cytokines, which will be discussed in further detail in subsequent sections of this review, also present barriers to APC activity in CNS and non-CNS sites alike. Novel strategies aimed at augmenting anti-tumor immune responses at the level of tumor antigen presentation may therefore arise from discoveries made at both CNS and non-CNS sites. Notably, DC phenotypes can also vary greatly depending on tissue type, raising the possibility that DC-based therapies may ultimately also require tailored approaches that account for site-specific tumor biology.

## Part IV: Immunosuppressive Cytokines – IL-10 and TGF-β

Cytokines with powerful immunosuppressive properties, including TGF-β and IL-10, are known mediators of tumor proliferation, invasion, and immune evasion. As such, targeted blockades of immunosuppressive cytokines are an attractive approach to tumor immunotherapy both in the brain and the body. A major challenge of cytokine-directed immunotherapy, however, lies in the pleiotropic and often paradoxical immune-regulatory functions of these cytokines. Neither TGF-β nor IL-10 is purely immunosuppressive and pro-tumorigenic in its effects. Therefore, developing successful immunotherapies that target immunosuppressive cytokines requires site-specific considerations that pay heed to micro-environmental context and tissue-specific biology.

### Interleukin-10

Interleukin-10, arguably the most potent anti-inflammatory cytokine (185), is secreted by numerous cell types of the innate and adaptive immune system, including APCs and CD4+ T-helper cells, as well as malignant tumors of the brain and the body (186, 187). T-helper cells, monocytes, macrophages, and DCs are particularly important both as targets and actors of IL-10-mediated immunosuppression (155). Binding of IL-10 to its receptor (IL-10R) on DCs activates STAT3 transcription factor, which suppresses STAT-dependent signaling of inflammatory cytokines, IL-6, TNF-α, and IL-1B (188, 189); upregulates IL-10 secretion (190); and maintains DCs in an immature, tolerogenic state (155, 191). In macrophages, monocytes, and DCs, IL-10 also suppresses antigen-presenting capabilities by activating MARCH1, an E3 ligase that ubiquintinates cell-surface MHC Class II molecules for endocytosis and destruction (192, 193). IL-10 also hinders cytotoxic T-lymphocyte effector functions by inducing and sustaining FoxP3 transcription factor expression in immunosuppressive Treg cells (194, 195).

Paradoxically, IL-10 can also exert pro-inflammatory and anti-tumor effects (42). In fact, IL-10 gene was first isolated from T-cells that also secreted IFN-γ (196), illustrating the complex relationship between anti-inflammatory and pro-inflammatory response of IL-10. IL-10 is a potent stimulator of NK cells (197), mast cells, and B cells, and, often in combination with other cytokines, can potentiate cytotoxic activity of CD8+ T cells (198–202). IL-10 also exerts important anti-angiogenic effects by suppressing cytokine promoters of angiogenesis, which in certain pre-clinical tumor models has been shown to inhibit tumor growth (41, 203).

To date, investigations into the role of IL-10 in tumor growth has largely focused on its immunosuppressive actions. However, both immunosuppressive and anti-tumor effects appear to be active in tumors at all sites to varying degrees (185), which naturally presents challenges for IL-10-directed immunotherapy.

#### IL-10: Malignant Gliomas

Human gliomas have long been known to produce IL-10 *in vivo* (204). Among subclasses of human astrocyte tumors, the most aggressive tumors contained the highest levels of IL-10 mRNA, with glioblastoma tissue containing the most of any astrocyte tumor (204). Rather than secreting IL-10 directly, however, glioma cells produce soluble factors that induce tumor-associated macrophages (TAMs) and microglia to secrete the majority of the cytokine (32).

Consistent with its immunosuppressive actions elsewhere in the body, glioma-associated IL-10 down-regulates MHC class II expression on monocytes and inhibits IFN-γ and TNF-α production by immune cells (36, 37). IL-10 also upregulates checkpoint molecule B7-H1 (PD-L1) on both glioma-associated macrophages and circulating monocytes in peripheral blood (103). B7-H1 can bind and stimulate PD-1 receptor on activated T cells, producing T-cell anergy and apoptosis (118, 205). Furthermore, IL-10 has been shown to confer growth advantages to glioma tissues. *Ex vivo*, IL-10 both increases glioma proliferation (206) and confers invasive potential to glioma cells in a dose-dependent manner (207).

In conjunction with other cytokines, IL-10 can also facilitate anti-glioma immune responses. Mice implanted with gliomas expressing both IL-10 and IL-2 had significantly smaller (99% smaller) tumor sizes and increased T-cell infiltration at 14 days post-implantation compared to mice with IL-10^−^/IL-2^−^ tumors (38). Additionally, this reduction in tumor size could not be reproduced with either IL-10 or IL-2 expressing tumors alone (38).

More recently, Vleeschouwer et al. reported that persistent and elevated IL-10 production by T-cells was in fact required for T-cell suppression of glioma growth following stimulation with tumor lysate-loaded dendritic cells (39). Ectopic IL-10 delivery during the T-cell stimulation phase further increased the levels of IFN-γ production and hindered tumor growth (39). It has been postulated that the complex interplay between IL-10 and IFN-γ might regulate the immunosuppressive effect of indolamine 2,3-dioxygenase (IDO) tryptophan metabolism by glioma-associated APCs, resulting in a stronger anti-tumor immune response (208). The role of IDO in glioma-induced immunosuppression is discussed in subsequent sections of this review.

#### IL-10: Non-CNS Tumors

While IL-10 also plays a duplicitous role in tumor suppression and progression at tissues outside of the CNS, its biological actions in peripheral sites also differ in several important ways. IL-10 mRNA and protein have been isolated from a variety of human tumors, including ovarian (209), breast (203, 210), renal cell (211), lung (212), squamous and basal carcinomas (213), and metastatic melanoma (33, 214). Unlike gliomas, however, where the vast majority of IL-10 is produced by tumor-associated macrophages and microglia, several peripheral tumors produce IL-10 directly. For example, metastatic melanoma (33) and bronchogenic carcinomas (34) produce IL-10 almost exclusively, with little or no secretion by TAMs.

At the same time, other peripheral tumors, similar to gliomas, may also rely upon TAMs to produce the majority of IL-10. HPV-16 associated carcinomas, for example, have been shown to recruit TAMs, which produce the majority of IL-10 (35). Whether or not similar soluble factors are utilized by gliomas and systemic tumors to induce TAMs to produce IL-10 is still unknown, but such knowledge would be therapeutically relevant for targeting IL-10 in these tumors.

In certain peripheral tumors, IL-10 also appears to have a particularly strong stimulatory effect on NK cells (197). In a murine B16 melanoma model, ectopic injection of IL-10 into the tumor mass reduced the numbers of infiltrating CD8+ and CD4+ T cells and macrophages (215), which is consistent with observations from gliomas; however, IL-10 also increased infiltration of NK cells in melanoma (215), which has not been reported in gliomas. Exogenous IL-10 was also shown to inhibit melanoma metastasis in mice that were deficient in B cells and T cells but with competent NK cells (41), suggesting that infiltrating NK cells may play a key role in suppressing metastatic spread.

The anti-angiogenic effects of IL-10 may also play an important part in inhibiting tumor growth and metastasis. IL-10 is known to suppress the macrophage production of pro-angiogenic cytokines, including IL-1, IL-6, IL-8, TNF-α, and MMP-9 (41, 216). Indeed, whereas the blood vessels were all but absent in the surrounding tissue of IL-10 secreting melanoma tumors, the tissue surrounding non-IL-10 producing tumors was highly vascularized (41). Whether IL-10 exerts similar anti-angiogenic and anti-metastatic effects in CNS tumors is yet unknown, although *in vitro* data suggest that the pro-proliferative effects of IL-10 in malignant gliomas may outweigh the inhibitory effects (206, 207).

Lastly, IL-10 serves a protective role in certain tissues of the body where chronic inflammation plays an etiological role in cancerogenesis. In these tissues, IL-10 is a key cytokine for maintaining anti-inflammatory T-regulatory cells and suppressing pro-inflammatory IL-17-expressing Th17 cells (217). Mice that were deficient in IL-10 spontaneously developed inflammatory bowel disease (IBD), which later progressed to colorectal carcinoma (43). Likewise, a small human study reported that IL-10 and IL-10R deficiencies, which has been linked to early onset IBD (45, 218), may also be associated with the development of malignant lymphomas (44). These pro-tumorogeneic associations become particularly important in the context of therapeutic approaches that may systemically deplete, or block the effects of, IL-10.

#### IL-10 in the Brain and Body: Therapeutic Implications

Taken together, these data illustrate the enigmatic role of IL-10-mediating tumor growth as well as suppression, the balance of which is greatly influenced by tumor biology and micro-environmental cues. It is particularly interesting that in the setting of malignant gliomas, IL-10 derived from TAMs exerts an overall tumorogenic and immunosuppressive effect, whereas IL-10 secreted in persistent and high levels by T-cells can produce pro-inflammatory and anti-tumor effects. These data indicate that cell of origin of IL-10 may determine, at least in part, its phenotypic actions in the tumor environment. Specific cell populations may therefore be selectively depleted to achieve the desired pro-inflammatory or anti-inflammatory effect.

From a therapeutic standpoint, it is also important to elucidate how IL-10 might interact with other cytokines in the microenvironment to generate an anti-tumor or pro-tumor response. IL-2, for example, appears to potentiate the anti-tumor response in malignant gliomas. In non-CNS tumors, IL-10 has been shown to augment CD8+ T-cell cytotoxicity in a manner that is dependent on its expression of IFN-γ and granzymes (219). Pegylated IL-10 (PEG-IL-10), which in pre-clinical tumor models was shown to expand tumor-resident CD8+ T cells and mediate tumor rejection (217), has entered human trials as monotherapy or in combination with chemotherapy for patients with advanced solid tumors, which include melanoma, NSCLC, renal cell, colorectal, ovarian, prostate, and pancreatic cancers (Clinical Trial NCT02009449) (Bauer 2014 ASCO). Whether PEG-IL-10 alone or in combination with IL-2 holds promise for treating malignant gliomas remains to be seen.

### TGF-β

TGF-β is a 25-kDa cytokine that is produced by several cell types, including both immune cells and malignant tumors (220). TGF-β is formed as a pre–pro-polypeptide and is activated through a series of protealytic cleavage steps. The active isoforms of TGF-β, TGF-B1, TGF-B2, and TGF-B3, signal by bringing together two pairs of serine/threonine kinases known as type I and type II TGF-β receptors (57). Canonically, cross-phosphorylation of type I and II receptors leads to downstream phosphorylation of Smad family of transcription factors, which migrate to the nucleus and regulate transcription of various target genes (57).

TGF-β is highly pleiotropic, regulating a wide array of biological functions that include cell proliferation, migration, survival, angiogenesis, embryonic stem cell differentiation, and immune surveillance (220). Its role in cancer genesis is also manifold, serving as a suppressor of early-stage tumor proliferation but an abettor of late-stage tumor progression (58). Elevated expression of TGF-β and its receptors by several human cancers, both in the brain and the body, has been associated with higher tumor grade and/or poorer prognosis (221). These malignancies include prostate cancer, small cell lung carcinoma, pancreatic cancer, gastric cancer, transitional cell carcinoma of the bladder, as well as malignant gliomas (221).

#### TGF-β: Malignant Gliomas

TGF-β was, in fact, initially isolated from the serum of patients with malignant gliomas. Fittingly described as a soluble “humoral immunosuppressive” factor, glioma-derived TGF-β significantly depressed lymphocyte functions and induced systemic lymphopenia, particularly in CD4+ T helper cell populations (222). Subsequent decades of research have further elucidated that TGF-β actually depresses cytotoxic functions of all cells of the immune system, facilitating immune evasion and glioma growth (52). MHC class II expression on glioma cells, macrophages, and microglia, for example, are significantly depressed by TGF-β (223). Expression of NKG2D activating receptor on the surface of NK cells are likewise reduced, as is production of CD8+ CTL cytolytic gene products perforin, granzyme A, granzyme B, FasL, and IFN-γ (224, 225). TGF-β also polarizes T-cells and monocyte-lineage cells toward immunosuppressive phenotypes, which further perpetuates a tolerogenic state that favors tumor growth (57). Moreover, TGF-β is believed to facilitate glioma growth and invasion by promoting angiogenesis (53), sustaining glioma stem cell populations (54), inducing the production of platelet-derived growth factor (PGDF), which serves as an autocrine proliferative signal for glioma cells (55), as well as increasing the synthesis of pro-invasive matrix metalloproteinases (56).

Strategies that block TGF-β signaling have been shown to restore anti-tumor immunity in pre-clinical glioma models. For example, *in vitro* silencing of TGF-β1 and TGF-β2 synthesis in human glioma cells using small interfering RNA (siRNA) techniques was shown to prevent NKG2D down-regulation on NK cells and enhance MICA expression on glioma cells (224). Furthermore, siRNA-silenced glioma cells displayed increased susceptibility to immune cell lysis (224). In a murine glioma model, inhibiting TGF-β1 receptor using SX-007, an oral serine/threonine kinase inhibitor, produced greater numbers of long-term survivors (33%) in the experimental group compared to control group (6%) (226). The treatment group receiving SX-007 also had higher levels of CD8+ T-cells in the CLNs than control groups, indicating TGF-β blockade can reverse its immunosuppressive effects (226). Taken together, these data illustrate that TGF-β confers predominately immunosuppressive and pro-invasive advantages to malignant gliomas, and blocking TGF-β signaling can reverse its malignant effects.

#### TGF-β: Therapies for Malignant Gliomas

In the brain, modulating TGF-β is particularly attractive. Radiation, a therapeutic cornerstone for malignant CNS tumors, has been shown to increase TGF-β expression both *in vitro* and *in vivo*. Neutralizing TGF-β might not only counteract the immunosuppressive and pro-invasive effects of TGF-β on the tumor but also attenuate the radiation-induced activation of TGF-β. Indeed, a small-molecule TGF-βR1 kinase inhibitor LY2109761 increased radio-sensitivity of GBM cell lines and stem cells *in vitro*. In combination with radiotherapy, LY2109761 reduced the tumor growth and prolonged the survival in ortho-topic intracranial murine glioma models compared to radiotherapy alone (227). Conceivably, this benefit might also extend to tumors at other sites that are frequently treated with radiotherapy, such as prostate adenocarcinoma or head-and-neck squamous cell carcinomas.

Several compounds targeting TGF-β signaling in malignant gliomas have entered clinical trials (220); their efficacy, however, remains inconclusive. One of the most promising compounds was Trabedersen, an anti-sense oligonucleotide against TGF-β2 mRNA that was shown to inhibit tumor proliferation and enhance anti-tumor immunity *in vitro* (228). In phase I/II trials, Trabedersen was associated with improved survival in patients with refractory high-grade gliomas compared to literature data (229). Although a subsequent randomized phase IIb clinical trial of Trabedersen reported improved tumor control and trended toward improved 2-year survival among patients with refractory anaplastic astrocytoma compared to chemotherapy (230), the results of the trial have been called into question based on several methodological weaknesses (231). The Phase III trial of Trabedersen, which was halted in 2012 due to patient recruitment issues, was recently terminated in light of advances in neurosurgical and first-line standard of care for glioblastoma (220). However, phase I and II trials of LY2157299, an oral TGF-β receptor kinase inhibitor, for newly diagnosed and recurrent glioblastomas have recently completed accrual, and efficacy data are expected in 2015 (232–235).

#### TGF-β: Non-CNS Tumors

Whereas TGF-β exerts predominately immunosuppressive and tumorigenic effects in the context of gliomas, its role in influencing tumor growth in other sites of the body is arguably more pleiotropic and context-dependent, which makes modulating TGF-β in systemic tumors exceedingly complex. Neutralizing TGF-β may indeed cause tumor regression at sites that depend on TGF-β for proliferation but, at the same time, may also inadvertently cause tumor growth in tissues where TGF-β serves as a tumor suppressor (50). TGF-β, for example, is a potent inhibitor of epithelial cell proliferation (236–238), and inactivating mutations of TGF-β receptors are implicated in the development of several human carcinomas (50). Neutralizing the protective effects of TGF-β could conceivably promote malignant transformation of epithelial tissue.

Even among tumors of the same tissue type, inactivating mutations of TGF-β and/or its receptor can lead to disparate effects. Mutations in TGF-β receptor are frequently found in colon cancer (239–242), and mouse models have shown that inactivating mutations in the TGF-β gene increases spontaneous formation of colorectal carcinoma (243). Yet, paradoxically, patients with a form of hereditary colorectal carcinoma, termed HNPCC, and who frequently have TGF-β receptor mutations actually have better prognoses than patients with sporadic colon cancer without TGF-βR mutations (239, 244). Lastly, similar to the potential off-target effects of IL-10, whether or not a tumor arises in a pro-inflammatory or anti-inflammatory environment also becomes a key consideration in modulating TGF-β. Gastric adenocarcinomas, for example, which can develop as a result of protracted tissue inflammation following *H. pylori* colonization, may flourish in the absence of TGF-β and other immunosuppressive cytokines.

Nevertheless, several strategies for targeting TGF-β in non-CNS tumors, including anti-sense oligonucleotides, monoclonal antibodies, vaccines, and small-molecule inhibitors, have shown moderate success in pre-clinical models of breast, colorectal, pancreatic, hepatocellular, and renal cell carcinomas, with some proceeding toward human trials (220). The efficacy as well as off-target effects of modulating this multi-faceted cytokine remain to be seen.

## Part V: Indolamine 2,3-Dioxygenase 1 – Tryptophan Metabolism

Indolamine 2,3-dioxygenase 1 is a cytosolic enzyme produced by macrophages and dendritic cells, primarily in response to pro-inflammatory factors (such as IFN-γ, IFN-α, IFN-β, and LPS) (245, 246). IDO catalyzes the rate-limiting step of tryptophan degradation, producing, among other Trp metabolites, kynurenine, which exerts several immunosuppressive effects that may help to regulate inflammation (66). Most notably, kynurenine facilitates expansion of T-reg populations and inhibition of T cell effector functions (67, 68). IDO, however, is also expressed by several human tumors in the brain and the body, including lung, prostate, colorectal, pancreatic, and endometrial cancers, as well as glioblastoma multiforme (60, 247). Moreover, level of IDO expression by malignant tumors has been correlated with poorer prognoses (248), indicating that IDO and the expansion of Treg populations may play a critical role in abetting tumors in evading host immunity.

### IDO: Malignant gliomas

Indolamine 2,3-dioxygenase 1 is not expressed in the brain under normal physiological conditions. IDO mRNA, however, is substantially elevated in human glioma tissues and correlates negatively with overall survival (60, 61). Similar to other tumor sites outside of the CNS, the malignant effect of IDO on glioma progression appears largely to result from IDO-mediated accumulation of thymus-derived nTreg cells, which subsequently exert immunosuppressive effects on effector cells in the tumor microenvironment (60). Specifically, production chemokine CCL22 by glioma cells is believed to play a key role in recruiting and trafficking peripheral nTregs into the glioma milieu, a subject that is discussed in more depth in subsequent sections of this review in Ref. (71, 72).

Recent data also indicate that glioma cells, rather than TAMs, microglia, and DCs, directly produce the majority of the IDO (60), which is distinct from tumors outside of the CNS where DCs account for the majority of tumor-derived IDO (62–65). In a murine glioma model where GL261 cells were injected intracranially into the brain of WT or IDO-deficient mice, peripheral expression of IDO had no impact on intratumoral T-cell accumulation or overall survival (60) between the two groups of mice. By comparison, implantation of IDO-producing GL261 tumor cells into the same set of mice resulted in significantly increased intratumoral Treg accumulation and reduced overall survival (60). Interestingly, when IDO-expressing and IDO-non-expressing glioma cells were implanted concurrently in separate cerebral hemispheres within the same mouse, any survival benefit normally attributed to IDO-deficient tumors was eliminated by the presence of IDO-expressing gliomas in the contralateral cerebral hemisphere (249). Taken together, these data illustrate that IDO, produced directly by glioma cells, globally suppresses the anti-glioma immune response by recruiting thymus-derived nTregs.

### IDO: Site-specific considerations for immunotherapy

Although tumors both in the brain and the body can exploit IDO-mediated immunosuppression to overcome host anti-tumor immune responses, molecular inhibition of IDO activity has produced different responses in different organs, which may reflect unique tissue-specific factors.

In a murine breast tumor model, 1-methyl-tryptophan (1-MT), a widely studied inhibitor of IDO, failed to inhibit tumor growth (250); however, in combination with cytotoxic chemotherapies, including paclitaxel, cisplatin, cyclophosphamide, and doxorubicin, 1-MT produced significant tumor regression (250). The synergic effects between cytotoxic chemotherapy and 1-MT have also been reported in melanoma models (251). In glioma models, however, it appears that, in sufficient doses, 1-MT alone can produce significant anti-tumor effects (249). Moreover, when co-administered with cytotoxic chemotherapy, 1-MT failed to improve survival over chemotherapy alone (249), suggesting that the synergism between IDO inhibition and chemotherapy may depend on differences in tissue biology between CNS and non-CNS tumors. It has been postulated, for example, that separate tryptophan-metabolizing enzymes, such as IDO2 or TDO, that are also known to mediate immunosuppression in gliomas, may provide compensatory kynurenine production under states of cellular stress (249).

Interestingly, administering 1-MT with anti-CTLA-4 and anti-PD-L1 monoclonal antibodies produced a 100% long-term survival rate in glioma-bearing mice (249), an improvement over the 90% long-term survival rate in anti-CTLA-4 and anti-PD-L1 therapies alone. By comparison, the same triple therapy regimen was dramatically less effective at extending survival in mice with intracranially implanted B16 melanoma tumors, illustrating that the utility of IDO modulation may differ substantially based on tumor type and environmental context (249).

Lastly, differential patterns of IDO expression among tumor types may also impact therapeutic efficacy of IDO modulation. Recent tissue analysis of 15 human tumor types showed that IDO expression was largely restricted to tumor cells, myeloid-lineage cells, and endothelial cells (62). The distribution of IDO expression among the three categories of cells, however, varied greatly from tumor to tumor. IDO expression within renal cell carcinomas tissue, for example, appeared to be largely restricted to the vasculature, whereas IDO expression within colorectal cancer tissue appeared to be limited to DCs (62). Whereas cervical tumor tended to express IDO on the outer edges of the parenchyma, IDO expression in endometrial tumors was more diffusely distributed throughout the parenchyma (62). The frequency of IDO expression also varied depending on tumor type. For example, cervical and endometrial carcinomas were found to be most frequently IDO+ (83 and 94% of all cervical and endometrial carcinoma tissue samples, respectively) while glioblastoma tissues were most frequently IDO− (only 8% of glioblastoma tissues were found to express IDO) (62). Further work is needed to characterize how variable expression patterns of IDO among different tissue types may affect IDO-targeted immune-modulation therapy.

## Part VI: T-Regulatory Lymphocytes

Regulatory T lymphocytes (Tregs) are a highly diverse and plastic subset of CD4+ immunosuppressive helper T cells that play an essential role in promoting immunological tolerance (252, 253). As guardians against autoimmunity, Tregs can also hamper anti-tumor immune responses and facilitate tumor growth, an undesired consequence of that has long been recognized (254, 255). Malignancies both in the brain and body actively recruit and sustain Tregs into the tumor microenvironment and parenchyma, and numerous studies have correlated higher intratumoral Treg density with higher tumor grades and poorer prognoses (256). Hence, Tregs are believed to play a pivotal role in tumor-mediated immunosuppression and subsequent immune escape, leading to the failure of immune therapies.

### Natural and adaptive Tregs

CD4+ Tregs comprise approximately 5–10% of circulating CD4+ T cell population, and, based on developmental origin, Tregs are classified as either thymus-derived natural Tregs (nTregs) or peripherally induced “adaptive” Tregs (iTregs) (257). Subsets of the CD8+ suppressive regulatory T-cells, for which less is known about their immunomodulatory roles in disease than CD4+ counterparts, also exist and are reviewed elsewhere (253).

nTregs develop in the thymus from CD4+ single-positive thymocytes via antigen presentation by thymic epithelial cells (257). nTregs characterized by stable and high-level expression of Forkhead Box P3 (FoxP3), key transcription factor and regulator for Treg development and immunosuppressive function (252). Mice and humans with rare FoxP3 gene dysfunctions suffer florid autoimmune attack on multiple organs and tissues, culminating in a fatal disorder known as immunodysregulation polyendocrinopathy enteropathy X-linked syndrome (IPEX) (258). More recently, the transcription factor Helios as well as neuropilin-1, a semaphorin III receptor, has also been identified as potential markers for nTregs (259–262). Although incompletely characterized, nTregs exert their immunosuppressive function in a contact-dependent, cytokine-independent mechanisms, which include the expression of surface molecules CTLA-4 and PD-L1, membrane-bound TGF-β, pericellular generation of adenosine, as well as through Granzyme B/Perforin and Fas/FasL pathways (49, 263–268).

By comparison, iTregs, which encompass several distinct CD4+ T cell types (257), differentiate in the periphery when antigens are presented to and recognized by naïve conventional CD4+ T cells (Tconv) under tolerogenic conditions. In contrast to nTregs, which display constitutive expression of FoxP3, iTreg FoxP3 expression is transient or even absent, and its induction appears dependent on IL-10 and/or TGF-β signaling (49, 252). iTregs also appear to exert their immunosuppressive effects by releasing soluble factors, such as IL-10 and TGF-β (49), instead of the cell-surface ligand molecules used by nTregs.

Ultimately, whether Tregs are thymically or peripherally derived, Tregs are capable of wholesale suppression of innate and adaptive effector immune cell function (253). From a therapeutic standpoint, however, it is valuable to understand the process by which tumor-infiltrating Tregs accumulate within various tumors, such that the targeted strategies might be developed to modulate specific Treg populations.

### Treg accumulation in brain

Greater numbers of glioma-associated Tregs has been associated with higher tumor grade (269), and levels of tumor-infiltrating Tregs may prove to be an important prognostic indictor for survival (270), though the data are conflicting (271–273). Based on current methods for evaluating Treg phenotype, the data suggest that tumor-infiltrating Tregs in malignant gliomas are predominately nTregs, rather than iTregs (69). In a murine glioma model, levels of tumor-infiltrating Tregs were significantly diminished for mice that were thymectomized prior to tumor implantation compared to that of non-thymectomized mice (69). In addition, over 90% of Tregs within the tumor expressed Helios transcription factor (69), which is known to be highly expressed on thymus-derived nTregs but not iTegs in mice and humans (262), strengthening the claim that glioma-associated Tregs may be nTregs.

The precise mechanism by which gliomas recruit nTregs is still under investigation; however, it is becoming evident that gliomas produce several soluble factors (72) that aid in recruiting nTregs into the microenvironment and parenchyma. In particular, gliomas are known to produce CC chemokine ligand 22 (CCL22), which serves as a potent chemotactic factor for leukocytes expressing CCL22 receptor CC chemokine receptor 4 (CCR4). Glioma-infiltrating Tregs express particularly high levels of CCR4 compared to other tumor-infiltrating lymphocytes (73), and several *in vitro* migration studies have demonstrated the ability of glioma-derived CCL22 to induce Treg chemotaxis (71–73).

CC chemokine ligand 2, another chemokine produced by human gliomas (74) and a weaker ligand for CCR4, has also been implicated in glioma-mediated Treg chemotaxis (71, 75). *In vitro* administration of blocking antibodies to CCR4 as well as CCL2 receptor, CCR2, arrested Treg migration toward glioma supernatant (71). Whether CCL22 and/or CCL2 are significant Treg chemotactic factors *in vivo* is still a matter of contention (71, 72, 75); however, it is evident that other soluble factors within the glioma microenvironment also contribute to Treg chemotaxis, and these factors remain to be identified (72).

Interestingly, outside of the tumor parenchyma and microenvironment, circulating CCL22 appears to be depressed in the sera of patients with malignant gliomas (274). Additionally, in a serum analysis of 1,208 patients with glioma, one group recently reported that lower serum levels of CCL22 were a negative prognostic indictor for overall survival (274). Because gliomas are known to exert global immunosuppressive effects, the lower levels of CCL22 in sera seen in patients with higher grade gliomas compared to lower grade gliomas was thought to reflect glioma-mediated suppression of peripheral APCs, which are the predominant producers of CCL22 *in vitro* and *in vivo* (275). The precise mechanisms underlying this relationship, however, remain to be elucidated. Further clarification is needed regarding whether glioma-related production of CCL22 is related to levels of CCL22 in peripheral blood, as well as whether glioma-derived CCL22 is also associated with disease prognosis.

Though iTregs may likely play a lesser role in glioma immunoresistance (69), there is reason to believe that gliomas are also capable of converting Tconv into iTregs *in vivo*. TGF-β and IL-10, both of which are produced by gliomas *in vivo*, have been shown to induce Treg conversion *in vitro* (252, 276). Prostaglandin E2, which is also produced by gliomas via cyclo-oxygenase 2 (COX-2), can induce *de novo* Tconv to Treg conversion (277, 278).

### Treg accumulation at non-CNS sites

Whether tumor-infiltrating Tregs in peripheral sites are thymus-derived or peripherally induced Tregs is also controversial, especially in the absence of definitive markers for distinguishing nTregs from iTregs (257). There is compelling evidence, however, that intratumoral Tregs from non-CNS sites also comprised predominately nTregs rather than iTregs, similar to the distribution in malignant gliomas. Using a mouse fibrosarcoma tumor model, Waight et al. recently demonstrated that intratumoral Tregs bore CpG hypomethylation at FoxP3 Treg-specific demethylated region (TSDR) (70), which is thought to be an epigenetic hallmark for nTregs (279). Moreover, epigenetic analysis of intratumoral Tregs from human NSCLC and ovarian tumors revealed demethylation at the FoxP3 TSDR similar to that observed in murine tumor models, suggesting that tumor-infiltrating Tregs in human tumors may also be nTregs (70). Whether similar distributions of Treg subtypes based on epigenetic markers are found in other peripheral tumors remains to be determined.

Similar to gliomas, several non-CNS tumors, including ovarian (76), breast (77), prostate (78), gastric (79), esophageal (80), as well as Hodgkin lymphoma (81) tumor cells can also elaborate CCL22 to help recruit Tregs into the tumor microenvironment. Notably, in one recent study of 417 cases of invasive breast cancer, high tumor expression of CCL22 was associated with higher histological grade and greater density of tumor-infiltrating Tregs (280). Furthermore, higher CCL22 expression was reported to be an adverse predictor of progression-free and overall survival (280). Higher ratio of stromal CCR4+ Tregs to CD8+ Tregs was also negatively associated with overall survival in human oral squamous cell carcinoma (OSCC) (281), indicating a potential relationship between CCL22 and overall survival in OSCC.

At the same time, Treg chemoattractant profiles can also vary greatly from tumor to tumor in peripheral sites. For instance, CCL17, another ligand to CCR4, does not appear to play a role in Treg chemotaxis in glioma or ovarian carcinoma but is a key mediator in Hodgkin’s lymphoma and gastric adenocarcinoma (81). In colorectal carcinoma, TAMs secreting CCL20 attracted tumor-infiltrating Tregs that highly expressed CC chemokine receptor 6 (282). Likewise, in an experimental melanoma model, tumor-infiltrating Tregs expressed CCR5 and preferentially migrated toward its ligands CCL3, CCL4, and CCL5, which were elaborated by tumor-infiltrating myeloid-derived suppressor cells (MDSCs) (82). Similarly, CCR5–CCL5 signaling also appears to play a prominent role in Treg migration in both human and murine pancreatic adenocarcinoma (83).

Though, as with the brain, the most recent data suggest that the majority of tumor-infiltrating Tregs in peripheral sites are also nTregs, non-CNS tumors can also elaborate TGF-β, IL-10, and PGE2, which can induce peripheral iTreg conversion. Administration of anti-TGF-β antibody *in vitro* blocked conversion of Tconv to the Treg phenotype, and *in vivo* administration of anti-TGF-β antibody in mice implanted with renal cell carcinoma reduced tumor burden, decreased numbers of circulating FOXP3+ CD25+ CD4+ cells in peripheral blood, and removed the immunosuppressive capabilities of FOXP3+ CD25+ CD4+ T cells (283). This leaves open the possibility that iTregs play an important but poorly understood role in tumor immunoresistance. In fact, one murine sarcoma model illustrated that intratumoral nTregs and iTregs may collaborate to suppress different arms of the adaptive immune response, with nTregs preferentially suppressing CD8+ T cells and iTregs suppressing CD4+ T cells, respectively (284).

### Therapeutic implications

From a therapeutic standpoint, these findings are particularly important. Traditional approaches to depleting Tregs, such as anti-CD25 antibodies (285) and cyclophosphamide (286), are largely non-specific, and whether these strategies preferentially target nTreg or iTreg populations is currently unknown (257). However, with the current knowledge that nTregs may comprise the majority of tumor-infiltrating Tregs in the brain and the body, it may be possible to devise targeted depletion strategies for nTregs, thereby minimizing side effects associated with indiscriminate systemic Treg depletion (258). For example, nTregs are believed to exert their immunosuppressive effects predominately via contact-dependent, cytokine-independent mechanisms (49). These include co-stimulatory and co-inhibitory molecules CTLA-4 and PD-L1, membrane-bound TGF-β, pericellular generation of adenosine, and granzyme B/perforin and Fas/FasL pathways (49, 263–268). Therefore, it may be possible to modulate nTreg activity by blocking the interactions between these immunosuppressive cell-surface ligands and their receptors (287–289).

Blocking Treg recruitment may offer another route for reducing intratumor Treg burden in a specific manner. CCL22–CCR4, a shared chemokine pathway for Treg migration in several tumors of the brain and the body, may prove useful for reducing Treg burden in a targeted manner (290). Recently, Adeegbe et al. reported that using anti-CCR4 antibodies in human melanoma patients selectively depleted CCR4+ Tregs while sparing naïve Tregs (257). Other strategies that interfere with the CCL22–CCR4 axis have demonstrated moderate success in *in vitro* and pre-clinical *in vivo* experiments (290–292).

Finally, within tissues of the body where inflammation promotes carcinogenesis, such as with gastric cancers or colorectal carcinoma, greater numbers of Tregs suppress inflammation and may therefore have anti-tumor effects. Greater degree of Treg tumor infiltration has in fact been associated with better prognosis in colorectal cancers (293, 294). Therefore, immunotherapeutic strategies that target Treg depletion need to consider the environmental context within which tumorigenesis occurs.

## Part VII: Tumor-Associated Myeloid Cells

The role of myeloid-lineage cells in promoting tumor growth and invasion has come into focus in recent years. At least five distinct subpopulations of tumor-associated myeloid cells (TAMCs) have been identified, including monocyte-derived tumor-associated macrophages (TAMs); angiogenic monocytes; immature, immunosuppressive myelomonocytic cells known as MDSCs; tumor-associated neutrophils (TANs); as well as microglia within the CNS (87). Their expansive roles in facilitating immunosuppression, angiogenesis, cellular proliferation, and tumor invasion in CNS and non-CNS sites have prompted investigations into new immunotherapeutic strategies aimed at neutralizing TAMCs. Representative classes of TAMCs as they relate to gliomas will be discussed below; an in-depth review of TAMCs can be referenced here (87).

### TAMCs in malignant gliomas

Microglia and monocyte-derived macrophages (i.e., TAMs) together account for the majority of glioma-associated myeloid cells (87, 159). Microglia, the resident macrophages of the CNS, compose 5–20% of the total glial cell population (84, 85) and play an essential role in the innate defense system of the brain (91). Monocyte-derived macrophages, by comparison, are normally restricted to the perivascular, choroid, and meningeal locations of the CNS (see Part II: The CNS Immune Environment), gaining entry to the parenchyma only after disease and/or inflammation have disrupted the integrity of BBB. In the setting of glioma, TAMs and microglia can comprise upward of 30% of the total tumor mass, with reports indicating that high-grade gliomas tend to exhibit greater levels of TAMs and microglia accumulation than low-grade gliomas (87, 295, 296).

Similar to monocyte-derived macrophages, both microglia and TAMs can embody pro-inflammatory (M1) as well as immunosuppressive (M2) phenotypes depending on environmental cues (99, 297). In the presence of inflammatory signals, classically activated microglia and macrophages skew toward an M1-like phenotype, characterized by increased capacity to migrate, phagocytose, secrete cytotoxic factors, as well as express MHC class II and co-stimulatory molecules for T cell activation (91). In the setting of gliomas, however, the data suggest that microglia and TAMs polarize toward an M2-like phenotype (91–93), particularly in late stages of disease progression (298), and exhibit immunosuppressive, pro-invasive properties that facilitate tumor growth. It is important to note that the M1/M2 classification is useful for illustrating the dichotomous role of microglia and TAMs in tumorogenesis but is ultimately an oversimplification, as TAMs and microglia exhibit a continuum of phenotypes at any one time, and the functional outcome may ultimately hinge upon the balance of pro-inflammatory and anti-inflammatory TAMs and microglia in the tumor microenvironment (159).

Recent work has produced convincing evidence that microglia and TAMs represent distinct classes of mononuclear phagocytic cells based on developmental origin (299, 300); however, distinguishing between TAMs and microglia in glioma tissue has proved difficult. Historically, cell-surface markers, CD11b integrin and common leukocyte antigen CD45, have been used to parse the two cell populations, with microglia expressing CD11b^high^/CD45^low^ and TAMs expressing CD11b^high^/CD45^high^, but the reliability of these markers in practice remains controversial (87). Newer genetic techniques employing inducible gene reporters to identify unique developmental markers in non-diseased murine models have had success in distinguishing monocyte-derived macrophages from microglia *in vivo* (301–303); however, whether such techniques can accurately identify macrophages and microglia in the setting of glioma remains to be determined. Therefore, the subsequent discussion will refer collectively to both macrophage populations as glioma-associated microglia and macrophages (GAMs) (304).

### Glioma-associated microglia and macrophages

Gliomas recruit GAMs in significant numbers, with GAMs comprising as much as one-third of all tumor-associated inflammatory cells (305). GAMs are recruited via glioma-derived chemo-attractants, including CCL2 (306), CCL7 (307), CX3CL1 (308), and stromal-derived factor-1 (SDF-1) (309); GAMs are subsequently sustained within tissue via glioma-derived growth factors, such as CSF-1, G-CSF, and hepatocyte growth factor (310–312). In exchange for pro-growth factors, GAMs provide the tumor with matrix metalloproteinases, which facilitate tumor growth and invasion (96), as well as tumor proliferation promoting factors, such as epidermal growth factor (EGF) (97) and vascular endothelial growth factor (VEGF) (98). Under the influence of glioma-associated cytokines, GAMs further upregulate immunosuppressive programmed death ligand 1 (PD-L1) (94, 95), which promotes T-lymphocyte anergy, as well as FASL, which promotes T-lymphocyte apoptosis (313, 314). Moreover, gliomas induce GAMs to substantially decrease the expression of MHC molecules and pro-inflammatory cytokines (TNF-α) while increasing the secretion of transcription factor, STAT3, likely through S100B-receptor for advanced glycation end produces (RAGE) axis (315). GAM STAT3 activation promotes the secretion of immunosuppressive cytokines, IL-6 and IL-10, which are known to inhibit cytotoxic T lymphocyte function, among other immunosuppressive actions (316, 317).

Discoveries surrounding the role of GAMs in promoting tumor growth have been followed closely by strategies to modulate their immunosuppressive actions. Transcription factor STAT3, which is upregulated in glioma-associated microglia, is a promising target for molecular intervention. *In vitro* blockade of STAT3 using siRNA reduced the microglial expression of immunosuppressive cytokines, IL-6 and IL-10 (316). *In vivo* silencing of STAT3 in a murine glioma model promoted a pro-inflammatory microglia response that inhibited tumor growth (316). Corosolic and oleanolic acids, known inhibitors of STAT3, have also been shown to reduce the macrophage expression of CD163, a marker of the immunosuppressive M2 phenotype, as well as IL-10, suggesting that these molecules may hold potential for reversing M2-like polarization of microglia (318, 319). Other novel approaches include the use of antibodies to block microglia chemotaxis toward gliomas, analogous to efforts aimed at attenuating Treg cell recruitment. Anti-CCL2 therapy, for example, has shown success in prolonging survival in murine glioma models (320). Other novel strategies have been well reviewed here (321).

### Myeloid-derived suppressor cells in malignant gliomas

Compared to TAMs and/or microglia, relatively less is known about the role of MDSCs in gliomagenesis and progression. MDSCs represent a diverse population of immature and highly immunosuppressive myeloid cells that accumulate in the tumor, blood, lymph nodes, and bone marrow of tumor-bearing hosts in response to tumor-derived factors, such as IL-6, IL-10, PGE2, TGF-β2, and VEGF (89, 322, 323). Though controversial, MDSCs are most commonly classified as either monocytic or granulocytic MDSCs (also known as polymorphonuclear MDSCs), with granulocytic MDSCs exerting weaker immunosuppression compared to monocytic MDSCs on a per cell basis (89). A population of pro-myelocytic MDSCs, representing an even more immature lineage of myeloid suppressor cells that are negative for both monocytic and granulocytic markers, has also more recently been described (324).

It is thought that granulocytic MDSCs suppress antigen-specific CD8+ T cell activity via production of reactive oxygen species (ROS), which, for example, could trigger apoptosis in activated T cells by decreasing Bcl-2 expression (325), while monocytic MDSCs increase l-arginine metabolism via NO and ARG-1 pathways, causing micro-environmental arginine depletion, ultimately leading to downregulation of T cell receptor components as well as T cell cell cycle arrest (326–328). Additionally, MDSCs are also thought to interfere with T-cell trafficking, induce NK- and T-cell anergy, and enhance Treg activation and expansion (329). In a study by Raychuadhuri et al., T cells isolated from patients with GBM had significantly depressed IFN-γ production following stimulation. Subsequent depletion of MDSCs from peripheral blood using anti-CD33/CD15-coated beads significantly restored T cell IFN-γ production *in vitro* (330).

In gliomas, the majority of circulating MDSCs appear to be predominately granulocytic (329). Interestingly, Gielen et al. recently reported that while patients with GBM contain elevated levels of both granulocytic and monocytic MDSCs in peripheral blood when compared to healthy controls, glioma tissues contain almost exclusively granulocytic MDSCs (331), a finding that may have important implications for MDSC-targeted therapy. In addition, the authors reported that patients who had had longer courses of dexamethasone for cerebral edema displayed greater levels of both classes of MDSCs in peripheral blood, a finding that could merely reflect patient-level differences in tumor mass but also possibly dexamethasone-mediated alterations to myeloid cell phenotypes, warranting further investigation (331). There is also compelling data suggesting that circulating MDSCs may arise from glioma-associated monocytes. Chae et al. recently showed that mice that received transgenic green fluorescent protein (GFP)+ CD11b+ splenic monocytes along with GL261-Luc cells not only had shorter survival, faster tumor growth, and higher levels of intratumoral and circulating MDSCs compared to mice that received GL261-Luc cells alone but also their work showed that 30–50% of circulating MDSCs were GFP+, suggesting that MDSCs arose directly from GFP+ monocytes (332).

Myeloid-derived suppressor cell-targeted immunotherapy is an area of active research. For example, various murine glioma models have shown that depletion of MDSCs, either via COX-2 inhibition (278), antibody-mediated MDSC depletion (278), or CCL2 neutralization (320), can prolong the survival. Other strategies for modulating MDSCs have been highlighted here (329).

### Tumor-associated myeloid cells in non-CNS sites

Immunosuppressive myeloid cells are not unique to CNS tumors and are equally important facilitators of tumor growth and invasion in peripheral sites as well. Higher density of tumor-associated macrophages (TAMs) has been associated with poorer prognosis in several human cancers, including breast, prostate, bladder, colorectal, and gastric cancers (333). Increased levels of M2-polarized TAMs have been correlated with accelerated metastasis and reduced survival in pancreatic (334) and renal cell carcinoma (88) as well as certain lymphomas (335). Indeed, several glioma-derived chemokine mediators that are important in re-purposing microglia with immunosuppressive functions are also implicated in polarizing peripheral tumor-associated macrophages toward an M2 immunosuppressive phenotype (99).

Although the relative distribution of microglia and TAMs in gliomas has yet to be fully characterized (see above discussion), an intriguing observation that the majority of tumor-infiltrating mononuclear phagocytes in murine gliomas may represent monocyte-derived macrophages rather than native microglia suggests that monocyte-derived macrophages may play a significant role in coordinating glioma growth (86). Immunotherapy aimed at modulating macrophage populations in the CNS may therefore be highly pertinent to managing immunosuppressive macrophages within non-CNS tumors, and vice versa.

Myeloid-derived suppressor cells have also been implicated in facilitating local and systemic immunosuppression in the setting of non-CNS tumors, including breast, colon, lung, kidney cancer, and head-and-neck cancers (89, 90), making MDSC-targeted therapy relevant to tumor immunotherapy at all sites. The mechanisms by which MDSCs arise and confound anti-tumor immunity, however, may differ depending on tumor site. For example, tumor-conditioned media from certain non-CNS tumors has been shown to induce immunosuppressive phenotypes in myeloid cells (322, 336); however, *in vitro* data from Rodrigues et al. revealed that direct contact between monocytes and glioma cells was needed to induce an MDSC-like phenotype in monocytes (90). Moreover, Rodrigues et al. failed to find a correlation between serum levels of tumor-derived cytokines known to stimulate MDSC proliferation in patients with gliomas compared to healthy counterparts (90), suggesting that the elevated levels of circulating MDSCs in patients with gliomas may arise from direct contact between tumor-infiltrating macrophages and/or monocytes and glioma cells rather than via systemic cytokine-induced conversion. Recent work from Chae et al., who showed GFP+ monocytes co-injected with GL261 cells into murine brains, led to increased levels of GFP+ MDSCs lends credence to the theory (332).

The relative proportions of circulating granulocytic, monocytic, and lineage-negative MDSCs may also vary depending on tumor type. Compared to patients with melanoma, renal cell carcinoma, and bladder carcinoma, patients with GBM had the greatest levels of granulocytic MDSCs (330). While the relative distribution of granulocytic, monocytic, and lineage-negative MDSCs in the peripheral blood of patients with renal cell carcinoma and bladder cancer appear consistent with that of GBM (i.e., granulocytic > lineage-negative > monocytic MDSCs), patients with melanoma have nearly equal percentages of granulocytic and lineage-negative MDSCs (330). The exact clinical relevance of differing proportions of MDSCs in different tumor types has yet to be elucidated; however, given that different subclasses of MDSCs may utilize different mechanisms of immunosuppression, MDSC-targeted immunotherapy may ultimately need to account for the predominant subsets of MDSCs associated with various tumor types.

Lastly, in keeping with the theme of other immunotherapeutic targets discussed in this review, targeting MDSCs may ultimately be highly contextual and tumor-dependent. Although depletion of MDSCs in certain glioma models has led to survival benefits (278, 320, 337), eliminating MDSCs may produce opposite effect in other tumor models. For example, Kerkar et al. reported that IL-12 immunotherapy in a B16 murine melanoma model “reprogramed” MDSCs, which in turn actually potentiated the anti-tumor effects of CD8+ T cells (338). By comparison, IL-12 immunotherapy prolonged the survival in a GL261 murine glioma model regardless of whether MDSCs were depleted (339), indicating that MDSCs may play a different supporting role in IL-12 immunotherapy in melanomas versus gliomas. Further work is needed to ascertain the functional outcome of depleting MDSCs in different tumor models. Simultaneously, it will also be prudent to assess the viability of “reprograming” MDSCs into mature myeloid cells that promote tumor elimination, similar to what has been accomplished with using all-trans retinoic acid in the treatment of acute pro-myelocytic anemia.

## Part VIII: Immune Checkpoints Molecules

Therapeutic modulation of co-stimulatory and co-inhibitory receptors of the immune system, often referred to as “immune checkpoint molecules,” has erupted in recent years following the seminal work in blockading cytotoxic T-lymphocyte antigen 4 (CTLA-4), a co-inhibitory molecule expressed on activated T-cells and Treg cells. Ipililumab, an mAb directed against CTLA-4, was the first therapy to procure survival benefits for patients with metastatic melanoma (340), providing proof-of-concept that disrupting checkpoint molecules could alone reverse tumor immunoresistance and lead to immune-mediated tumor eradication.

Numerous immune checkpoint molecules have subsequently been identified (341) and hold substantial promise as targets for tumor immunotherapy in the brain and the body. In this regard, characterizing immune checkpoint molecule, programmed cell death protein-1 ligand (PD-L1), its role in immune regulation, and opportunities for therapeutic intervention in the CNS and other sites is particularly instructive.

### Programmed cell death protein-1 ligand

Programmed cell death protein-1 ligand (B7-H1, CD274) is a trans-membrane glycoprotein of the B7 family of co-stimulatory molecules with potent immune-regulatory properties (120, 342). Under normal physiological states, PD-L1 is largely restricted to myeloid-lineage cells, including DCs and macrophages (343), and binds its receptor, programmed cell death protein-1 (PD-1, CD279), which is predominately expressed on activated T-cells (342, 344). Activation of PD-1 suppresses proliferation and lytic functions of effector T cells while expanding immunosuppressive Treg cells (341). Under inflammatory states, PD-L1/PD-1 signaling protects against rampant T cell activation and autoimmunity. Numerous tumors in the brain and the body, however, also express PD-L1, which can suppress tumor-directed cytotoxic T-cells that would otherwise destroy it (341). PD-L1 expression in several tumors, including renal cell carcinoma (105, 106), lung carcinoma (107, 108), breast carcinoma (109), and glioblastoma (119), has been correlated with higher tumor grade and poorer prognosis (120).

### PD-L1: Malignant gliomas

Aside from endothelial cells of the BBB, PD-L1 is usually not expressed in the CNS (345–347), and PD-L1 expression on glial cells and/or neurons is typically signs of pathological states. In gliomas, PD-L1 expression is positively correlated with malignancy grade (205) and is likely driven by genetic alterations that also potentiate oncogenesis. Loss of PTEN tumor suppressor gene enhances the expression of PD-L1 on glioma cells, suggesting that activation of PI(3)K-Akt-mammalian target of rapamycin (mTOR) pathway may modulate PD-L1 translation (101). Concurrently, greater degrees of PD-1 expression on peripheral CD4+ and CD8+ T cells are also observed in gliomas of higher malignant grade (119), and co-culturing alloreactive CD4+ and CD8+ T cells with PD-L1-expressing glioma cells significantly depresses the production of inflammatory cytokines, such as IFN-γ and IL-2 (118). Accordingly, the PD-1/PD-L1 axis has become an attractive target for glioma immunotherapy. Blocking PD-L1 on glioma cells with mAbs in combination with radiotherapy has yielded particularly potent survival benefits in pre-clinical models (348).

In addition to glioma cells, TAMCs provide another source of PD-L1. Microglia are known to upregulate PD-L1 expression under inflammatory states (94), and microglial expression of PD-L1 has indeed been reported in human glioblastoma tissue (102). Recently, Parsa et al. reported that gliomas also induce PD-L1 expression on tumor-infiltrating macrophages, which may further contribute to PD-1-mediated T-cell suppression (103). This finding is particularly compelling, as it provides a cellular basis by which gliomas may induce global immunosuppression via monocyte-derived macrophages. Whether the benefits observed from anti-PD-L1 mAbs are due primarily to blocking PD-L1 expressed by gliomas, TAMs, microglia, or combinations thereof, remains to be determined.

Notably, in the first study that extensively characterized the role of neurons in the GBM microenvironment in inhibiting tumor growth, Liu et al. reported that high levels of neuronal PD-L1 expression in tumor-adjacent brain tissue (TABT) corresponded favorably with overall patient survival, and low TABT PD-L1 expression and high GBM PD-L1 expression portended poorer patient survival (104). Mechanistic analysis revealed neurons expressing PD-L1-induced caspase-mediated apoptosis of GBM cells (104). Neuronal expression of IFN-β, which enhances neuronal expression of PD-L1, was also postulated to suppress glioma growth via its tumor-suppressor functions (104). These findings illuminate the potential drawbacks of indiscriminate administration of PD-L1 neutralizing antibodies, which might limit native host neuron defenses against gliomagenesis.

### PD-L1: Non-CNS tumors and implications for therapy

The biological pathways implicated in enhancing PD-L1 expression in gliomas are equally important to the development of immunoresistance in tumors outside of the CNS. Expression of PD-L1 on colorectal carcinomas, for example, has also been linked to loss of PTEN tumor suppressor (349). Similarly, PI3K/mTOR pathway activation is also associated with PD-L1 expression in breast (115), lung (108), renal cell (106), and prostate carcinomas (116). Molecular therapies targeting these pathways are therefore relevant to all of these tumors.

At the same time, other malignancies also utilize distinct signaling pathways to enhance PD-L1 expression, requiring that targeted molecular suppression of PD-L1 be tailored toward each site based on tumor-specific biology. For example, MyD88/TRAF6 and MEK/ERK pathways enhance PD-L1 expression in multiple myeloma (117), while constitutive activation of anaplastic lymphoma kinase (ALK) drives PD-L1 expression in certain lymphomas and lung carcinomas (341, 350).

Targeting the PD-L1/PD-1 axis by indirectly modulating PD-L1-bearing TAMs may also prove to be relevant strategy for CNS and non-CNS tumors alike (100, 110). Hepatocellular carcinomas, like gliomas, also recruit high numbers of PD-L1-expressing TAMs into the tumor microenvironment, corresponding with poor prognosis (111).

Lastly, the astonishing discovery that neuronal expression of PD-L1 in the tumor microenvironment protects against gliomagenesis (104) may also find parallels in the body. Pancreatic, heart, endothelial, small intestine (112), and placental tissues (113) also express PD-L1 (114). Whether tissue expression of PD-L1 at sites outside of the CNS similarly protects against local tumorogenesis remains an open question.

### Beyond PD-L1

Beyond PD-L1, effector immune cells express myriad checkpoint molecules that also contribute tumor immunoresistance both in brain and body. Targeting cytotoxic T-lymphocyte antigen 4 (CTLA-4), which modulates early stages of T-lymphocyte activation, has proved useful in reversing immunoresistance in gliomas, as it has in non-CNS tumors (351–355). In an experimental glioma model, anti-CTLA-4 mAb procured an 80% long-term survival rate, concurrent with enhanced proliferation of CD4+ CD25− T cells and resistance to suppression by Treg cells (351). Glucocorticoid-induced tumor necrosis factor receptor-related gene (GITR) has emerged as an important checkpoint molecule in Treg cells (356). Additionally, Tim-3 (357) and 4-1BB (CD137) (358) are other key immune checkpoint molecules that will also require site-specific considerations, especially as immunotherapeutic strategies develop to target these ligands individually and in combination (68).

## Part IX: Concluding Remarks

A major challenge for the field of brain tumor immunology lies in elucidating key determinants and constituents of the pro-inflammatory and anti-inflammatory responses in the CNS such that they might be augmented for therapeutic gain. Contrary to the historical view of the CNS as immunologically sequestered from the rest of the body, the immune responses in the CNS are linked and complementary to immune processes in the periphery. The phenotype of the immune response often hinges upon cytokines and cellular mediators that exert highly pleiotropic and sometimes paradoxical actions depending on the specific tumor and environmental context. In evaluating these processes, however, it will be helpful to recognize that routes by which the CNS coordinates immune modulation are not without precedent: analogous immunological mechanisms exist at sites outside of the CNS, and advances in tumor immunotherapy in peripheral sites may therefore illuminate novel approaches to brain tumor immunotherapy, and vice versa (1). Therefore, this suggests that the intricacies of the brain immune environment need to be examined within the context of the entire body.

## Conflict of Interest Statement

The authors declare that the research was conducted in the absence of any commercial or financial relationships that could be construed as a potential conflict of interest.
